# What Drives Fast Food Consumption in Asian Low‐ and Middle‐Income Countries?—A Narrative Review of Patterns and Influencing Factors

**DOI:** 10.1002/puh2.70095

**Published:** 2025-08-04

**Authors:** Rafid Hassan, Abu Ahmed Shamim, Masum Ali, Md. Ruhul Amin

**Affiliations:** ^1^ Institute of Nutrition and Food Science University of Dhaka Dhaka Bangladesh; ^2^ Nutrition Research Division, International Centre for Diarrhoeal Disease Research, Bangladesh (icddr,b) Dhaka Bangladesh; ^3^ Center for Non‐Communicable Diseases and Nutrition, BRAC James P Grant School of Public Health BRAC University Dhaka Bangladesh; ^4^ Poverty, Gender, and Inclusion, International Food Policy Research Institute (IFPRI) Dhaka Bangladesh

**Keywords:** fast food, low‐ and middle‐income countries (LMICs), Asia

## Abstract

Fast food has become a common dietary choice worldwide, with significant health consequences. In low‐ and middle‐income countries (LMICs), particularly in Asia, the consumption of fast food has risen, yet research providing a comprehensive summary of fast food consumption patterns is limited. Therefore, this review consolidates evidence on the patterns and factors influencing fast food consumption in Asian LMICs. A comprehensive literature search was conducted using PubMed, Google Scholar, and references of relevant studies, covering peer‐reviewed articles published in English from January 1, 2011, to June 30, 2023. A total of 87 studies met the inclusion criteria, encompassing data from 178,554 individuals across 26 countries. The findings indicated a higher fast food consumption with a preference for Western fast food, such as pizza, burgers, fried chicken, French fries, and sandwiches, over local options. Key factors driving fast food consumption included taste, affordability, accessibility, mass media advertisement, restaurant environment, service quality, and family/peer influence. Furthermore, socioeconomic status, age, gender, and educational background influenced the consumption. For adults, factors like time constraints, lack of alternatives, employment status, health consciousness, stress, and food quality and hygiene were important. The increasing presence of fast food in the Asian diet highlights the need for comprehensive policies to curb this trend and protect public health.

## Graphical abstract

This review highlights the high prevalence and frequent consumption of fast food, particularly western items such as pizza, burgers, fried chicken, French fries, and sandwiches, among populations in Asian LMICs. Factors influencing fast‐food consumption include taste, affordability, availability, mass media advertising, restaurant environment, service quality, family and peer influence, socioeconomic status, demographic characteristics (age, gender, education, employment), limited time for cooking, lack of healthy alternatives, health consciousness, stress, and concerns about food quality and hygiene.

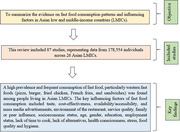

## 1 Introduction

The dietary landscape of Asian countries has undergone a remarkable transformation over recent decades, marked by a shift from traditional diets to the adoption of the Western diet. This transition is characterized by an increased consumption of fast food, animal products, oil, sugar‐sweetened and processed food, and ultra‐processed food [1, 2, 3]. Such dietary shifts are further reinforced by the rapid proliferation of fast food establishments across Asia [3], contributing significantly to the nutrition transition observed not only in developed nations but also in low‐ and middle‐income countries (LMICs) [4].

The term “fast food” is loosely defined, originating from its introduction by Merriam‐Webster, which defines it as food that can be swiftly prepared and served [5]. This category encompasses a range of mass‐produced food items tailored for rapid preparation and distribution, commonly retailed by restaurants, concession stands, and convenience stores [6]. According to the USDA, fast food restaurants are establishments where customers make payment for their food before it is served [7]. Furthermore, fast food is defined by the Foodbook study as food prepared in restaurants that do not offer table service but instead offer counter‐service, drive‐through, or carry‐out alternatives [7]. Fast food is one of the largest components of the food industry. The rapid growth of fast food in Asia can be attributed to various factors, such as unregulated food marketing, technological advancements in the food industry, globalization, urbanization, trade liberalization, and economic progress [3, 8, 9, 10, 11]. Originating from the United States, the fast food culture has transcended borders to become a global phenomenon, even reaching smaller towns in Asia. This expansion is not solely driven by multinational giants like McDonald's, KFC, and Pizza Hut, but also by domestic enterprises imitating the products and operational models of their foreign counterparts [3]. The international appeal of fast food is also evident in the success of chains like McDonald's and Yum!, which generate significant sales overseas, with 65% and 50% of their sales coming from international markets, respectively [5].

Fast food is characterized as larger in portion size, with palatability that typically contains higher levels of refined carbohydrates, sugars, fats, saturated fats, trans fats, cholesterol, and salt, along with a lower level of essential micronutrients and dietary fibers [12, 13, 14, 15]. Due to its high energy density and glycemic load, fast food consumption has been associated with weight gain and obesity [16]. Notably, overweight/obesity are key risk factors for a range of non‐communicable diseases (NCDs), including cardiovascular diseases, various metabolic syndromes, liver disease, osteoarthritis, sleeping disorders, certain types of cancers, and mental illness, contributing to a greater economic burden [17, 18]. The increasing trend of overweight/obesity has become a serious issue in LMICs [19]. This region contains about half of the world's stunted and overweight children, experiencing the double burden of malnutrition [20], and one out of every three LMICs is struggling with the double burden of malnutrition [21]. In Asia, the highest number of stunted and overweight children has been observed, exacerbating the situation [20]. Higher fast food consumption leads to a higher health burden. The consumption frequency can be a predictor of their diet and health status, as higher consumption of fast food is related to higher BMI [22] and lower diet quality [13].

In Asia, there is a scarcity of comprehensive reviews on fast food consumption. Although two reviews have covered certain Asian countries, none have specifically focused on LMICs in Asia, encompassing both children/adolescents and adults [23, 24]. Therefore, this review aims to gather scientific evidence to provide insights into fast food consumption patterns and their key driving factors in Asian LMICs.

## 2 Methods

### 2.1 Literature Search Approach

Fast food is defined differently by various sources. In Asia, although many street foods or junk foods are considered fast food, not all of them are classified as such [5]. Therefore, a comprehensive search was conducted in PubMed, which was chosen due to its extensive coverage of public health and nutrition‐related studies. To supplement this, Google Scholar was searched for gray literature, and the reference lists of relevant articles were screened to capture additional studies. The search strategy utilized a combination of MeSH and non‐MeSH terms, including fast food, junk food, Western food, ultra‐processed food, street food, unhealthy food, and unhealthy diet, as these terminologies were closely related to defining fast food (Table S1).

### 2.2 Inclusion and Exclusion Criteria

This review included original, peer‐reviewed, full‐text primary articles written in English and published between January 1, 2011, and June 30, 2023, focusing on LMICs in Asia. The studies included in this review met the following criteria based on the PECO framework:

- **Population (P)**: Individuals from LMICs in Asia;
- **Exposure (E)**: Consumption of fast food;
- **Comparison (C)**: Not applicable;
- **Outcome (O)**: Fast food consumption frequency or patterns, preferred or commonly consumed fast food items, and influential factors behind consumption.

Interventional studies, qualitative studies, and reviews were excluded. All identified studies were categorized into two groups based on age: children/adolescents and adults. Studies that did not specify the age or could not be categorized into either age group were excluded.

### 2.3 Data Extraction and Analysis

All records from the databases were downloaded and uploaded to EndNote for title and abstract screening. All selected abstracts were then evaluated for inclusion criteria, and key information from the included studies was extracted using an Excel data extraction table. The extracted data included author, publication year, location, participants, study design, sample size, gender, age, sample characteristics, fast food consumption frequency, influencing factors, and preferred fast food items.

### 2.4 Definitions

For the fiscal year 2023, the World Bank classified 33 countries as LMICs in Asia based on gross national income: Afghanistan, Bangladesh, Bhutan, Cambodia, China, India, Indonesia, Iran, Iraq, Jordan, Kazakhstan, Laos/Lao PDR, Lebanon, Malaysia, Maldives, Mongolia, Myanmar, Nepal, Pakistan, Philippines, Russian Federation, South Korea, Sri Lanka, Syria, Tajikistan, Thailand, Timor‐Leste, Turkey, Turkmenistan, Uzbekistan, Vietnam, West Bank, and Yemen [25].

## 3 Results

**FIGURE 1:**
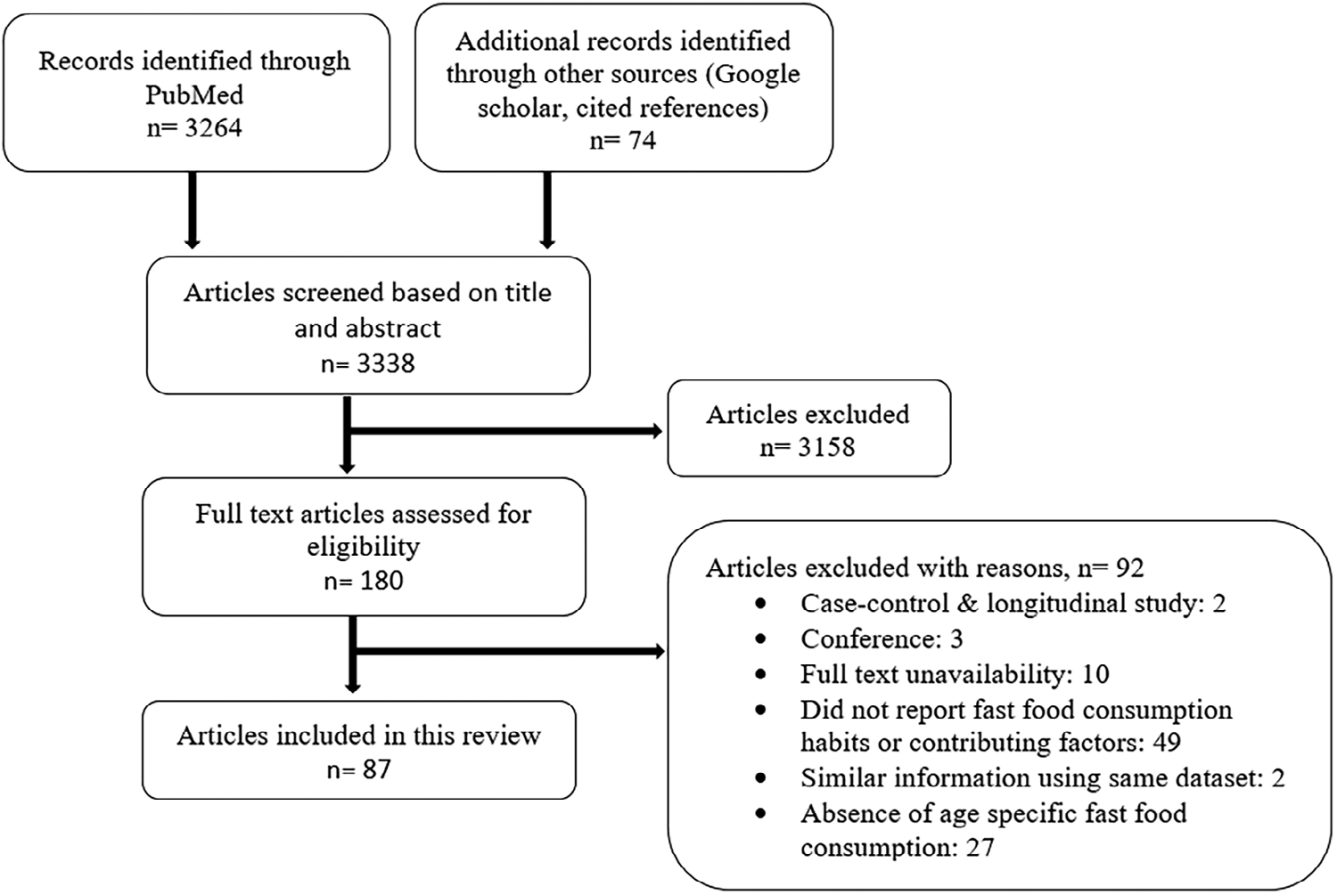
Flowchart of the study selection procedure.

A total of 3338 potential studies were identified in the electronic databases. After screening titles and abstracts, a total of 180 studies were considered for full‐text review. However, 92 of these did not meet the inclusion criteria. Thus, a total of 87 studies were included in this review, among which two were multi‐country studies. A summary of the process is detailed in Figure 1, and an overview of the 87 identified studies can be found in Table S2. These studies covered 26 countries from 33 LMICs in Asia, extracting information for 178,554 individuals.

### 3.1 Fast Food Consumption Frequency

The studies examined fast food consumption across various timeframes, including daily, weekly, and monthly intervals. The findings showed significant regional variations in consumption patterns, with frequent consumption reported.

#### 3.1.1 Fast Food Consumption Frequency Among Children/Adolescents

Fast food consumption among children and adolescents was widespread across Asian LMICs, though the frequency varied significantly by country, age, gender, and socioeconomic status. Studies from India, Pakistan, Nepal, and Bhutan indicated particularly high consumption rates. In India, approximately 98% of school‐going students consumed fast food, with 85% doing so regularly and over 20% consuming it more than three times per week [26, 27, 28, 29, 30]. A similar trend was observed in Pakistan, where nearly 96% of school‐aged children consumed fast food weekly [31], and 54% were frequent consumers [32]. In Nepal, three‐fourths of adolescents consume fast food weekly, with one‐third consuming it daily [33], whereas in Bhutan, around 90% consume fast food weekly [34].

Fast food consumption appeared to increase with age in several countries, including Malaysia, Iraq, and Bangladesh. In Malaysia, fast food intake was significantly higher among adolescents, with rates ranging from 48% to 83%, whereas younger children consumed fast food far less frequently [35, 36, 37, 38]. In Iraq, half of the primary school students consumed fast food weekly [39], a figure that rose to 57% among adolescents [37]. A similar pattern was observed in Bangladesh, where 68% of college‐going adolescents consumed fast food weekly, compared to 54% among younger school‐aged adolescents [37, 40].

In Iran, fast food consumption varied widely depending on socio‐demographic factors. Although some studies reported consumption rates as high as 70% among adolescents [41], others found much lower rates (15%–27%) [42, 43, 44]. Interestingly, in some Iranian studies, sweet snacks were preferred over fast food, particularly among girls [43]. Westernization of dietary patterns shaped fast food consumption in China, where 52% of children and adolescents consumed Western fast food weekly, whereas 44% preferred local Chinese fast food options [45].

In other countries, weekly fast food consumption among school‐going adolescents was also high: Thailand (82%) [37], Sri Lanka (43%–80%) [46, 47], Lebanon (77%) [47], Timor‐Leste and Afghanistan (65%) [37], Indonesia (56%) [37], Mongolia (55%) [37], the Philippines (49%) [37], and Syria (43%) [37]. However, consumption was considerably lower in the Maldives, Yemen, and Vietnam, where weekly consumption was around 35% [37, 47].

Gender differences in fast food consumption varied across countries. In Pakistan, high school and college girls consume more frequently than boys [48], whereas among younger children, boys tend to have higher consumption [49]. Similar trends were observed in Iraq [50], China [45], Bangladesh [51], Syria [52], and Vietnam [53], where boys consumed more than girls. However, in Malaysia, girls consumed more fast food than boys [38] (Table 1). Deatils of the consumption patterns can be found in Table S3.

**TABLE 1:** Fast food consumption frequency among children and adolescents.

Country	Fast food consumption frequency
Afghanistan	1–3 days/week: 52.2%, 4–7 days/week: 10.8%, ≥1 times/week: 65%, mean day/week: 2.4 [37]
Bangladesh	Once/week: 53.2%–68.3%, 1–2 days/week: 28%, 1–3 days/week: 35.9%–42.9%, ≥3 days/week: 26%–64.1%, mean day/week: 2.4, male consumed more than female [37, 40, 51]
Bhutan	Weekly consumption: 90.4% [34]
China	Weekly consumption: 32.4%, Western fast food/week: 51.9%, Chinese fast food/week: 43.6%, mean consumption/week: 0.6 to 1.5, male was frequent consumer than female [37, 54, 55, 56]
India	Once/week: 28.8%–62.7%, 1–2 times/week: 44.2%, 2 times/week: 15.4%–90%, 1–3 times/week: 62.6%, ≥2 times/week: 21.9%–57.5%, ≥3 times/week: 38.8%–90.4%, 4–7 times/week: 22.4%, occasionally: 17%, fast food preference and habit: 98% [26, 27, 28, 29, 30, 57, 58]
Indonesia	1–3 days/week: 45.3%, 4–7 days/week: 10.5%, ≥1 times/week: 56.5%, mean day/week: 2.3 [37]
Iran	Daily: 2.4%–10.6%, once/week: 22.7%–69.8%, 1–2 items/week: 24.2%, 2 times/month: 26.9%, 1–3 times/week:12.5%, ≥3 times/week: 2.7%–10%, monthly: 26.3%–43.9%, seasonally: 13.8%, yearly: 10.8% [41, 42, 43, 44, 59]
Iraq	Once/week: 34.6%–56.5%, 1–3 days/week: 45.3%, 2–3 times/week: 12.3%, >3 days/week: 10.5%–37% (male: 37%, female: 25%), daily: 1.8%, mean day/week: 2.3–3 (male: 3, female: 2.4) [37, 39, 50]
Maldives	≥1 times/week: 35.1% [47]
Mongolia	1–3 days/week: 37.7%, 4–7 days/week: 17.4%, ≥1 times/week: 55.1%, mean day/week: 2.6 [37]
Nepal	Once/week: 22.2%, 2 times/week: 24.8%, >2 times: 19.3%–89.6%, ≥1 times/week: 75.3%, daily: 10.3%–33.8%, monthly junk foods: 60.3% [33, 47, 60]
Pakistan	1–2 times/week: 29.5%–72.9%, 1–3 times/week: 44%–46%, ≥3 times/week: 13.4%–23.3%, daily: 5.4%–8%, frequent consumer: 54%, occasionally: 57%, mean consumption/week: 2.1 times for male and 2.5 times for female, everyone consumed fast food [31, 32, 48, 49, 61]
Philippines	1–3 days/week: 47.8%, 4–7 days/week: 4.1%, ≥1 times/week: 49%, mean day/week: 1.9 [37]
Sri Lanka	≥1 times/week: 42.8%, once/week: 60%,1–2 times/ month: 20%, daily: 20% [46, 47]
Syria	1–3 times/week: 37.8%–85.8%, 4–7 days/week: 5%–14.2%, ≥1 times/week: 42.5%, mean day/week: 1.8, male consumed more frequently than female [37, 52]
Thailand	1–3 days/week: 36.8, 4–7 days/week: 43.3, ≥1 times/week: 81.8%, mean day/week: 4.1 [37]
Timor‐Leste	1–3 days/week: 55.4%, 4–7 days/week: 11.8%, ≥1 times/week: 64.9%, mean day/week: 2.4 [37]
Vietnam	1–3 days/week: 24.4%, 2–6 times/week: 1.6%, 4–7 days/week: 5.3%, ≥1 times/week: 30.3%, mean day/week: 1.7, male consumed more than female [37, 53]
Yemen	≥1 times/week: 34.5% [47]

#### 3.1.2 Fast Food Consumption Frequency Among Adults

The frequency of fast food consumption among adults varied widely across countries. Several studies reported high consumption frequencies in Southeast Asia, particularly among undergraduate students, with India, Bangladesh, Pakistan, Sri Lanka, Malaysia, and Vietnam showing strikingly high numbers. In India, university students reported higher consumption rates (81%–95%) weekly, with a significant proportion consuming it ≥3 times/week [62, 63, 64, 65, 66]. Similarly, in Bangladesh, 94% of university students preferred fast food and showed a clear preference for foreign fast food chains over local ones [67]. A wide variation in weekly fast food consumption among university students was also found, ranging from 54% to 98% in Bangladesh [68, 69, 70, 71]. Furthermore, social media users (e.g., Facebook) had higher fast food intake in Bangladesh [72]. Among Pakistan's university students, consumption rates range from 58% to 91%, with a significant portion of female students consuming fast food regularly [73, 74, 75, 76]. In Sri Lanka, 98% of university students consumed fast food weekly, and half of them consumed it daily [77]. In Malaysia, studies showed a wide range in consumption, from 23% to 99% [78, 79]. In Vietnam, 42% of university students consumed fast food 1–2 times/week [80].

Fast food consumption also followed a higher trend among university students in the Middle East, such as in Syria (74% weekly) [81], Jordan (59% ≥ 2 times/week) [82], Turkey [83], and the West Bank (83% ≥ 3 times/week) [84]. In Iran, weekly fast food consumption varied from 11% to 69% [85, 86, 87, 88, 89].

Among working adults, fast food consumption was generally lower compared to students. For example, in Cambodia, only 63% of working individuals reported consuming fast food in the past month [90], whereas in Malaysia, consumption was significantly lower in low‐income areas [91, 92]. South Korea showed the lowest fast food consumption rate, with only 10% consuming it regularly [93].

Gender disparities in fast food consumption were evident in several regions. In Bangladesh, Turkey, Iran, and the West Bank, males were more likely to consume fast food than females. However, in Malaysia, females tend to consume fast food more frequently than males (Table 2). Details of the consumption patterns are avialable in Table S4.

**TABLE 2:** Fast food consumption frequency among adults.

Country	Fast food consumption frequency
Bangladesh	Once/week: 40.8%–50.5%, 1–2 times/week: 24%–27%, ≥4 times/week: 4.6%, ≥1 times/week: 54%–98.5% (male: 56% and female: 44%), daily: 15%, once/month: 20.1%–25.2%, 2–3 times/month: 29%, 1–3 times/month: 44.5%–48.1%, ≥3 times/month: 35.2%, ≥4 times/month: 14%–45.6% [67, 68, 69, 70, 71, 72, 94, 95]. Fast food preference: 71%–94.2%, preference of foreign fast food restaurants 60.3% vs. local fast food restaurants 39.7% [67], male preferred fast food over female [69]
Cambodia	Monthly fast food consumption: 62.7% [90]
India	<1 time/week: 6.8%–10%, Once/week: 30.8%, 1–2 times/week: 42.8%–80%, 3–4 times/week: 13.3%–23.7%, ≥3 times/week: 19.6%–74.6%, daily: 19.1%–44.6%, occasionally: 18.1%–22.7% [62, 63, 64, 65, 66, 96]. 85%–100% consumed fast food [63, 96]
Iran	≥1 times/week: 10.7%–31%, ≥2 times/week: 50%–52%, once/month: 8%–15.9%, 1–2 times/month: 20.9%, 2 times/month: 11.8%–25.6%, 1–3 times/month: 29.1%, monthly fast food consumption: 72.4% (males: 80.7% vs. females: 67.4%), mean consumption/month: 2.7 times [85, 86, 87, 88, 89, 97, 98]
Jordan	≥2 times/week: 59.4% [82]
Malaysia	≥1 times/week: 15%–34% (male: 23.5 vs. female: 34%), 1–5 times/week: 99%, ≥4 times/month: 24.3%, <4 times/month: 75.7% [78, 79, 91, 92]. 93% preferred fast food [79]
Pakistan	≥1 times/week: 57.4%–91.2%, weekends: 26.7%, occasionally: 48.71% [74, 75, 76, 99], 64%–94% consumed fast food [73, 76]
South Korea	10% habituated to fast food [93]
Sri Lanka	Once/week: 11%–16.7%, 2–3 times/week: 21%, 4–6 times/week: 12%, daily: 54%, 1–2 times/month: 66.7%–83.3% [46, 77]
Syria	Once/week: 44.5%, 2 times/week: 17.7%, ≥3 times/week: 11.4% [81]
Turkey	Female vs. male–once/15 days: 40.6% vs. 38.6%, 3 times/week: 19.8% vs. 28.4%, 5 times/week: 11.5% vs. 5.6%, once/month: 28.2% vs. 27.3% [83]
Vietnam	1–2 times/week: 42%, 1–2 times/month: 27% [80]
West Bank	12 times/week: 30.9%, 3 times/week: 52.4%, male consumed more fast food than female [84]

#### 3.1.3 Factors Influencing Fast Food Consumption Among Children/Adolescents

Table 3 describes the factors that influenced children and adolescents to consume fast food. Taste, convenience, availability, peer influence, and media exposure emerged as dominant factors influencing fast food consumption in this study. However, regional differences were evident, with parental control and family culture playing a stronger role in Asian countries like India [100], China [101], Indonesia [102], and Iran [41].

**TABLE 3:** Factors influencing the consumption of fast food among children and adolescents.

Factors	Country	References	Factors	Country	References
Taste	Bangladesh, India, Iran, Pakistan	[31, 44, 51, 57, 103]	Urbanization	India	[103]
Accessibility or convenience	Bangladesh, China, Iran, Pakistan	[32, 44, 51, 101]	Inexpensive	Iran	[44]
Advertisements and media influence	India, Indonesia, Iran, Pakistan	[31, 41, 102, 103]	Evening meals with family members	India	[100]
Availability	India, Nepal	[60, 100, 103]	Habit	Iran	[104]
Parental control or mediation	India, Indonesia, Pakistan	[31, 100, 102]	Past behavior	Iran	[104]
Family and peer influences	Iran, Nepal	[41, 60, 104]	Ethnicity	Malaysia	[38]
Proximity of fast food outlets	Iran, Sri Lanka	[41, 44, 46]	Location of schools	Malaysia	[38]
Educational qualification	Bangladesh, Sri Lanka	[46, 51]	Public school	Nepal	[60]
Gender	Bangladesh, Malaysia	[38, 51]	Nuclear family	Nepal	[60]
Socioeconomic circumstances	Bangladesh, Iran	[44, 51]	Living with parents	Nepal	[60]
Educational level of caregiver	China, India	[100, 101]	At the time of traveling, reading	Nepal	[60]
Variety or diversity	India	[57, 103]	Friends outgoing or gathering	Pakistan	[31]
Quick service	India	[57, 103]	Lack of time to cook	Pakistan	[31]
Income	India, Pakistan	[31, 100]	Social and environmental enjoyment	Pakistan	[32]
Age	Malaysia, Sri Lanka	[38, 46]	Hunger and satiety	Pakistan	[32]
Ideology of caregiver	China	[101]	Employment status	Sri Lanka	[46]
Brand value	India	[103]			

In India, the variety of fast food items, brand value, quick service, family income, and urbanization were driving forces to consume fast food [100, 103]. Children and adolescents in Nepal who studied in public schools and lived in small families with parents consumed more fast food. Furthermore, they consumed fast food while traveling, reading books, or staying in school [60]. Lack of parental involvement in cooking had an impact in Pakistan [31]. In Pakistan, some children and adolescents consumed fast food when they were hungry or going out with friends [31]. In Iran, adolescents’ behavior, habits, and lifestyle were responsible for their fast food consumption [104]. In Malaysia, the age of children and adolescents, their ethnicity, and the location of the educational institution played an important role in consuming fast food [38].

#### 3.1.4 Factors Influencing Fast Food Consumption Among Adults

The key factors that explained the fast food consumption among adults were described in Table 4. In this study, taste, affordability, convenience, lack of time to cook, restaurant environment, and family/peer influences emerged as the most consistent drivers of fast food consumption. However, many other cultural, social, and economic factors influenced fast food consumption, which varied by region.

**TABLE 4:** Factors influencing the consumption of fast food among adults.

Factors	Country	References
Taste	Bangladesh, India, Indonesia, Iran, Sri Lanka, Turkey	[62, 65, 67, 69, 70, 77, 83, 88, 92, 95, 96]
Price	Bangladesh, Indonesia, Pakistan, Sri Lanka, Vietnam	[67, 70, 76, 77, 80, 92, 99]
Accessibility or convenience	Bangladesh, India, Pakistan, Sri Lanka, Thailand	[46, 62, 67, 70, 76, 106]
Lack of time to cook	India, Indonesia, Jordan, Pakistan, Turkey	[65, 78, 82, 83, 96, 99]
Environment of the restaurants	Bangladesh, Iraq, Pakistan	[67, 76, 99, 105]
Family and peer influences	Bangladesh, India	[69, 70, 95, 96]
Sociability	Bangladesh, Turkey	[70, 72, 83]
Food quality	Bangladesh, Iraq, Pakistan	[67, 99, 105]
Pocket‐friendly or inexpensive	Bangladesh, Iran, Turkey	[70, 83, 88]
Fun	Indonesia, Iran, Pakistan	[78, 87, 99]
Service quality	Bangladesh	[67, 72]
Availability	Bangladesh, Indonesia	[69, 92]
Quick service	Bangladesh, Iran	[70, 88]
Lack of alternatives	Bangladesh	[69, 70]
Habit	Bangladesh, Pakistan	[69, 99]
Friends outgoing or gathering	Bangladesh, Indonesia	[78, 95]
Recreational activity	Bangladesh, Iraq	[95, 105]
Stress relief	Bangladesh, India	[95, 96]
Family culture	Iran, Pakistan	[88, 99]
Advertisements and media influence	Iran, Iraq	[88, 105]
Health consciousness	Pakistan, Vietnam	[80, 99]
Income	Pakistan, Thailand	[99, 106]
Food safety and hygiene	Sri Lanka, Vietnam	[77, 80]
Age	Sri Lanka, Thailand	[46, 106]
Educational qualification	Sri Lanka, Thailand	[46, 106]
Employment status	Sri Lanka, Thailand	[46, 106]
Personal aspects	Bangladesh	[72]
Payment system	Bangladesh	[67]
Location	Bangladesh	[67]
Free Wi‐Fi facility	Bangladesh	[95])
Emotion	India	[62]
Variety or diversity	India	[96]
Home delivery services	India	[96]
Decoration of fast food	India	[96]
Tasteless hostel meal	India	[65]
Knowledge	Iran	[97]
Subjective norms	Iran	[97]
Brand reputation	Pakistan	[99]
Hunger and satiety	Pakistan	[76]
Gender	Thailand	[106]

In countries like Bangladesh and Pakistan, people consumed fast food out of habit or pleasure [69, 76, 78, 87, 99] and during social gatherings [70, 72, 78, 95]. Family culture played a role in developing fast food habits in regions like Iran, Malaysia, and Pakistan [78, 88, 99]. Furthermore, brand value and reputation were considerable reasons in Pakistan [99]. Among Bangladeshi adults, consumption was affected by a lack of alternatives to fast food [69, 70], availability of modern recreation facilities, Wi‐Fi, and mode of payment [95]. Some people consume fast food as a means of stress relief in India and Bangladesh [95, 96]. People living in Iran and Iraq were influenced by mass media advertisements [88, 105]. Besides, knowledge, subjective norms, perception of health benefits, and disorganized student life also played important roles in Iran [97]. People in Iraq were persuaded to take their kids to a fast food restaurant for entertainment. Playful surroundings and the availability of room for kids to play were therefore seen as important considerations [105]. Many Pakistanis ate fast food when they were hungry [76]. In Thailand, fast food consumption varied by age, educational level, and occupation type of the customer [106].

### 3.2 Commonly Consumed Fast Food Items in LMICs of Asia

Table 5 demonstrates the most commonly consumed fast food items in LMICs in Asia. Pizza, burgers, fried chicken, and sandwiches were widely consumed across most countries. Furthermore, biscuits, noodles, chips, cake, pastries, hotdogs, doughnuts, spaghetti, and sausages were frequently reported in India, Nepal, Cambodia, Vietnam, and Malaysia. Alongside these, country‐specific consumption patterns were also observed.

**TABLE 5:** Variety of consumed fast food items in LMICs of Asia.

Fast food items	Country	References
Pizza	India, Iran, Nepal, Pakistan, Bangladesh, Iraq, Malaysia, Turkey, Vietnam	[27, 30, 31, 44, 65, 66, 69, 76, 80, 83, 95, 96, 98, 100, 105, 107, 108, 109, 110, 111]
Burgers	India, Iran, Nepal, Pakistan, Bangladesh, Iraq, Malaysia, Turkey, Vietnam	[27, 30, 31, 65, 66, 69, 76, 80, 83, 88, 96, 100, 105, 109, 110, 111]
Fried chicken	India, Bangladesh, Pakistan, Iran, Malaysia, Vietnam	[30, 31, 69, 76, 80, 96, 98, 109]
Sandwiches	Iran, Pakistan, Malaysia, Vietnam	[31, 44, 76, 80, 88, 98, 109]
Noodles	India, Nepal, Bangladesh	[30, 33, 69, 100, 110]
Biscuits	India, Nepal, Cambodia	[33, 90, 100, 107, 110]
Chips	India, Nepal, Vietnam	[33, 80, 100, 110]
Momo	Nepal, India	[33, 65, 96, 110]
Chowmein	Nepal, India	[33, 65, 108, 110]
French fry	Pakistan, Malaysia	[32, 66, 76, 109]
Cake	India, Cambodia, Malaysia	[30, 90, 109]
Pastry	India, Malaysia, Sri Lanka	[77, 107, 109]
Samosa	India, Bangladesh	[27, 95, 100]
Hotdog	Iran, Cambodia, Malaysia	[44, 90, 109,]
Doughnut	Nepal, Malaysia, Cambodia	[90, 109, 110]
Sausage	Cambodia, Malaysia, Vietnam	[80, 90, 109,]
Spaghetti	Cambodia, Malaysia, Vietnam	[80, 90, 109]
Chocolate	India	[27, 107]
Cookies	India, Nepal	[100, 110]
Wafer	India, Bangladesh	[95, 100]
Pav bhaji	India	[100, 108]
Ice cream	Nepal, Malaysia	[109, 110]
Fries	Pakistan, Nepal	[109, 110]
Shawarma	Pakistan, Iraq	[31, 105]
Meat ball toast	Cambodia, Malaysia	[90, 109]
Patties	India, Sri Lanka	[77, 108]
Rolls	India, Sri Lanka	[77, 96]
Puffs	India	[30]
Indian sweet	India	[107]
Maggi	India	[107]
Kachori	India	[107]
Namkeen	India	[107]
Fried rice	India	[107]
Vada pav	India	[107]
Chinese bhel	India	[107]
Pakora	Nepal	[33]
Panipuri	Nepal	[33]
Candies	Nepal	[33]
Fried food	Nepal	[110]
Daalmoth	Nepal	[110]
Papad	Nepal	[110]
Grill	Cambodia	[90]
Roast	Cambodia	[90]
Steak	Cambodia	[90]
Pasta	Cambodia	[90]
Macaroni	Cambodia	[90]
Chole bhature	India	[108]
Golgappa	India	[108]
Biryani	India	[96]
Kebab	Iraq	[105]
Falafel	Iraq	[105]
Meat dough	Iraq	[105]
Nuggets	Malaysia	[109]
Waffle	Malaysia	[109]
Pie	Malaysia	[109]
Porridge	Malaysia	[109]
Wade	Sri Lanka	[77]
Fish bun	Sri Lanka	[77]
Lahmacun	Turkey	[83]

In Bangladesh, adults consumed noodles most frequently [69]. Cambodian adults usually consumed grilled pork, bacon, ham, roast, steak, meat ball, toast, pasta, macaroni, or spaghetti [90]. In India, traditional items such as chole bhature, kachories, pakoras, pav bhaji, panipuri, golgappa, biryani, and namkeen were popular [96, 107, 108]. Furthermore, sweets, Maggi, patties, rolls, and puffs were commonly consumed in India [27, 30, 96, 107, 108]. The most consumed fast food items among Iraqi adults were kebabs, shawarma, falafel, and meat dough [105]. Among Malaysian adults, ice cream, nuggets, meat balls, waffles, pie, and porridge were popular [109]. Popular fast food items among children/adolescents in Nepal include pakora, panipuri, ice cream, fried food, daalmoth, and fries [33, 110]. Furthermore, rolls, wade, fish buns, and patties were frequently consumed in Sri Lanka [77]. Vietnamese people prefer salad [80]. In Nepal and India, noodles, chocolate and candies, momo, and chowmein were also popular fast food items [27, 30, 33, 65, 100, 107, 108, 110]. Several studies in Bangladesh and India reported the consumption of samosas and wafers [27, 95, 100].

## 4 Discussion

This review is the first to offer a comprehensive overview of fast food consumption patterns in Asian LMICs over the past decade. The findings revealed a substantial rise in fast food consumption across two different age groups, highlighting a significant dietary shift in the region. Furthermore, this review identified the key driving factors influencing fast food consumption and delineated the spectrum of preferred fast food items.

The review identified that more than three‐quarters of children/adolescents frequently consumed fast food (at least once a week) in India, Pakistan, Nepal, Bhutan, Thailand, Lebanon, Syria, and Malaysia. Furthermore, the fast food consumption rate was found to be higher in the rest of the countries. These findings were corroborated by a study among adolescent girls in LMICs, where 75% reported weekly consumption [112]. However, this rate was slightly lower compared to a study across 54 LMICs, where around 55% of adolescents consumed fast food weekly, with a pooled prevalence of 57% in the Southeast Asia region [37]. Among adults, fast food consumption varied from 42% to 100% weekly in countries like Bangladesh, India, Pakistan, Sri Lanka, Malaysia, Jordan, Vietnam, Syria, and the West Bank. Similarly, higher consumption was noted among young adults in Kuwait, where 82% consumed fast food two or more times weekly [113], and in Singapore, where 71% indulged in Western fast food weekly [114]. In South Korea and China, consumption rates were lower compared to other LMICs. Despite a general trend toward increasing fast food consumption in China, its expansion is still limited [115]. This might be due to the implementation of laws and regulations and parental concerns about a healthy diet in China [10, 38] as well as the promotion the traditional Korean diet in South Korea [116].

This review found that the prevalence of fast food consumption was more pronounced among adults than children/adolescents. Several studies conducted in European countries and Australia have identified comparable trends, where takeaway food consumption was found to be increased from adolescence to adulthood, with high fast food consumption observed between the ages of 18 and 45 years [117, 118, 119, 120, 121]. Adults typically possess greater autonomy and income sources, which are positively associated with fast food consumption. Furthermore, their engagement in income‐generating activities, coupled with time constraints, often compelled them to consume fast food [22].

Several studies suggested that the most preferred fast food items were pizza, burgers, French fries, sandwiches, shawarma, fried chicken, kebabs, sausages, and noodles [4, 113, 122, 123, 124], and the findings presented here agree with this review. Though local fast food constituted a larger share of fast food items in India, Nepal, Sri Lanka, and Turkey, Western fast food was consumed more frequently. The increased consumption of Western fast food can be attributed to the rapid rise of modern food retail outlets, including supermarkets, restaurants, and convenience stores. Over the past 15 years in Asia and the Pacific region, these modern outlets have experienced growth rates ranging from 14% to 1243% across various countries. This expansion has driven a significant rise in the sales of ultra‐processed food [125].

The unparalleled economic growth and urbanization in Asian countries have reshaped living patterns, with half of the population now residing in urban areas [125]. The concurrent trends of trade liberalization and open markets have made unhealthy fast food more accessible. Even a healthy diet is costlier than an unhealthy diet in Asia [4]. Nearly 1.9 billion people, constituting 44% of the population in Asia and the Pacific, struggle to afford a nutritious diet, with Southern Asia facing an even more substantial challenge, with 70% of the population unable to afford a healthy diet. Consequently, a large portion of people rely on processed or highly processed foods due to their ready availability and cheaper nature [125]. Besides, millions of dollars are invested in Asia to popularize fast food [37]. Many driving factors are responsible for this kind of popularity of fast food. With consistent results, the literature suggested its lucrative features, including taste, appearance, convenience, accessibility, availability, and inexpensiveness, for this fast food culture [113, 126]. More women are involved in economic activities, and long working hours compel them to consume this convenient fast food due to limited time to cook [127]. However, advertisements, food marketing, and discounts on price influenced people to consume [12]. Although many people visited fast food restaurants to socialize with friends and family members [126]. Family income, restaurant milieu, hygiene, and food safety were also some concerning issues in choosing fast food restaurants [128].

In this review, some studies suggested gender variation in fast food consumption, whereas others did not. In agreement with the literature, our review found that males consumed more fast food in Bangladesh, China, Iraq, India, Iran, Jordan, Malaysia, Pakistan, Syria, Turkey, Vietnam, and the West Bank [117, 120, 129]. However, findings from other studies in India, Pakistan, and Malaysia showed that females consumed more. Whether the consumption among females is more or less than that of males, they are more susceptible to overweight, obesity, and micronutrient deficiencies. The increased risk to females is likely due to a sedentary lifestyle and lower basal metabolic rate compared to males [22].

The findings of this review highlighted the urgent need for policy interventions to address the growing fast food culture in LMICs. Governments should implement public health campaigns to raise awareness about the health risks associated with fast food consumption. Additionally, regulatory measures such as taxation on unhealthy food, subsidies for nutritious alternatives, and stricter marketing restrictions—especially targeting children and adolescents—could help mitigate the rising prevalence of diet‐related diseases [11, 37, 130]. Policies should also encourage food reformulation to reduce harmful ingredients and improve the nutritional profile of fast food items. Strengthening food safety regulations and monitoring the nutritional composition of fast food products and front‐of‐pack labeling to highlight unhealthy nutrient content can further support healthier food environments [130]. Given the increasing role of modern retail outlets and fast food chains, it is essential for policymakers to collaborate with the food industry to foster healthier eating environments. Urban planning strategies should also be designed to enhance access to affordable and nutritious food options. At the same time, workplace and institutional policies should encourage the availability of healthier meal alternatives, aligning with broader public health goals.

Despite the growing body of research on fast food consumption in LMICs across Asia, significant gaps remain. Most available studies are cross‐sectional, limiting the ability to establish causality between fast food consumption and health outcomes. Future research should prioritize longitudinal studies to assess the long‐term effects of fast food consumption on obesity, NCDs, and overall dietary patterns. Moreover, qualitative research is needed to explore the socio‐cultural and psychological factors driving fast food consumption. Insights into consumer motivations, as well as the role of peer and family influences, will help to design more effective behavioral change interventions. There is a lack of research examining the impact of government regulations on fast food consumption patterns. More research is needed to determine the impact of these interventions on consumer behavior and health outcomes. Additionally, limited data exist on the nutritional quality of commonly consumed fast food. Future studies should assess levels of trans fats, sodium, and added sugars to guide policy efforts aimed at improving food quality and ensuring compliance with regulatory standards.

A key strength of this review was its use of comprehensive search criteria to maximize the identification of relevant publications. However, limitations include the failure to adhere strictly to systematic review standards. Nevertheless, this study provides valuable evidence to guide policymakers in implementing regulations to curb the rise of fast food consumption in the region. Furthermore, the findings lay the groundwork for future research focused on developing innovative, health‐conscious approaches to fast food, addressing the underlying factors driving its consumption.

## 5 Conclusion

This review revealed widespread consumption of fast food in LMICs across Asia, with Western fast food like pizza, burgers, fried chicken, French fries, and sandwiches being particularly popular. Several key factors driving this consumption included taste, affordability, availability, mass media influence, restaurant environment, service quality, family and peer influence, socioeconomic factors, age, gender, education, employment status, time constraints, stress, and health consciousness. The evidence underscores a critical situation, as fast food consumption is linked to overweight and obesity. This situation is concerning, especially in these economically vulnerable regions. To address these challenges, the implementation of targeted governmental policies is crucial. Such measures will help curb the expansion of unhealthy fast food practices and mitigate their adverse health effects in this region.

## Author Contributions

**Rafid Hassan**: conceptualization (equal), data curation (equal), formal analysis (equal), methodology (equal), writing – original draft (equal), writing – review and editing (equal). **Abu Ahmed Shamim**: writing – review and editing (supporting). **Masum Ali**: writing – review and editing (supporting). **Md. Ruhul Amin**: conceptualization (equal), methodology (supporting), supervision (lead), writing– review, and editing (lead).

## Ethics Statement

The authors have nothing to report.

## Conflicts of Interest

The authors declare no conflicts of interest.

## Supporting information




**Supporting File 1:** puh270095‐sup‐0001‐Tables.docx

## Data Availability

Data sharing does not apply to this article, as no datasets were generated or analyzed during the current study.
