# Supplementary material for: What Drives Fast Food Consumption in Asian Low‐ and Middle‐Income Countries?—A Narrative Review of Patterns and Influencing Factors
Source: Public Health Chall. 2025 Aug 4;4(3):e70095. doi: 10.1002/puh2.70095 (PMC12320721; doi:10.1002/puh2.70095)
Supplement: Supplementary file 1 — Supporting File 1: puh270095‐sup‐0001‐Tables.docx [file PUH2-4-e70095-s001.docx]

**Supporting information**

**Supplementary Table 1** Search strategy.

|  | **Search terms** |
| --- | --- |
| 1^st^ terms  (Fields: Title/Abstract, Text Word, Mesh) | fast food* OR fast-food* OR junk food* OR western food* OR ultra-processed food* OR street food* OR unhealthy food* OR unhealthy diet* |
| 2^nd^ terms  (Fields: Title/Abstract, Text Word) | (Bangladesh* OR India* OR Pakistan* OR Nepal* OR Bhutan* OR Myanmar* OR Indonesia* OR Iran* OR Philippi* OR Sri Lanka* OR Tajikistan* OR Timor* OR Vietnam* OR Uzbekistan* OR West Bank OR Gaza OR Palestine OR Mongolia* OR Afghan* OR South Korea* OR Yemen* OR Cambodia* OR Chin* OR Iraq* OR Jordan* OR Kazakhstan* OR Lebanon* OR Malaysia* OR Maldiv* OR Thai* OR Turkey* OR Turkmenistan* OR Russia* OR Laos OR Lao PDR) |
| Combination of terms using Boolean operators | (("fast food*"[Text Word] OR "fast food*"[Text Word] OR "junk food*"[Text Word] OR "western food*"[Text Word] OR "ultra processed food*"[Text Word] OR "street food*"[Text Word] OR "unhealthy food*"[Text Word] OR "unhealthy diet*"[Text Word] OR ("fast food*"[Title/Abstract] OR "fast food*"[Title/Abstract] OR "junk food*"[Title/Abstract] OR "western food*"[Title/Abstract] OR "ultra processed food*"[Title/Abstract] OR "street food*"[Title/Abstract] OR "unhealthy food*"[Title/Abstract] OR "unhealthy diet*"[Title/Abstract]) OR ("fast foods"[MeSH Terms] OR "fast foods"[MeSH Terms] OR ("junk"[All Fields] AND "food"[MeSH Terms]) OR (("western"[All Fields] OR "westerns"[All Fields]) AND "food"[MeSH Terms]) OR "food, processed"[MeSH Terms] OR (("street"[All Fields] OR "street s"[All Fields] OR "streets"[All Fields]) AND "food"[MeSH Terms]) OR (("unhealthier"[All Fields] OR "unhealthiness"[All Fields] OR "unhealthy"[All Fields]) AND "food"[MeSH Terms]) OR (("unhealthier"[All Fields] OR "unhealthiness"[All Fields] OR "unhealthy"[All Fields]) AND "diet"[MeSH Terms]))) AND ("bangladesh*"[Text Word] OR "india*"[Text Word] OR "pakistan*"[Text Word] OR "nepal*"[Text Word] OR "bhutan*"[Text Word] OR "myanmar*"[Text Word] OR "indonesia*"[Text Word] OR "iran*"[Text Word] OR "philippi*"[Text Word] OR "sri lanka*"[Text Word] OR "tajikistan*"[Text Word] OR "timor*"[Text Word] OR "vietnam*"[Text Word] OR "uzbekistan*"[Text Word] OR "west bank"[Text Word] OR "Gaza"[Text Word] OR "Palestine"[Text Word] OR "mongolia*"[Text Word] OR "afghan*"[Text Word] OR "south korea*"[Text Word] OR "yemen*"[Text Word] OR "cambodia*"[Text Word] OR "chin*"[Text Word] OR "iraq*"[Text Word] OR "jordan*"[Text Word] OR "kazakhstan*"[Text Word] OR "lebanon*"[Text Word] OR "malaysia*"[Text Word] OR "maldiv*"[Text Word] OR "thai*"[Text Word] OR "turkey*"[Text Word] OR "turkmenistan*"[Text Word] OR "russia*"[Text Word] OR "Laos"[Text Word] OR "lao pdr"[Text Word] OR ("bangladesh*"[Title/Abstract] OR "india*"[Title/Abstract] OR "pakistan*"[Title/Abstract] OR "nepal*"[Title/Abstract] OR "bhutan*"[Title/Abstract] OR "myanmar*"[Title/Abstract] OR "indonesia*"[Title/Abstract] OR "iran*"[Title/Abstract] OR "philippi*"[Title/Abstract] OR "sri lanka*"[Title/Abstract] OR "tajikistan*"[Title/Abstract] OR "timor*"[Title/Abstract] OR "vietnam*"[Title/Abstract] OR "uzbekistan*"[Title/Abstract] OR "west bank"[Title/Abstract] OR "Gaza"[Title/Abstract] OR "Palestine"[Title/Abstract] OR "mongolia*"[Title/Abstract] OR "afghan*"[Title/Abstract] OR "south korea*"[Title/Abstract] OR "yemen*"[Title/Abstract] OR "cambodia*"[Title/Abstract] OR "chin*"[Title/Abstract] OR "iraq*"[Title/Abstract] OR "jordan*"[Title/Abstract] OR "kazakhstan*"[Title/Abstract] OR "lebanon*"[Title/Abstract] OR "malaysia*"[Title/Abstract] OR "maldiv*"[Title/Abstract] OR "thai*"[Title/Abstract] OR "turkey*"[Title/Abstract] OR "turkmenistan*"[Title/Abstract] OR "russia*"[Title/Abstract] OR "Laos"[Title/Abstract] OR "lao pdr"[Title/Abstract]))) AND (2011/1/1:2023/6/30[pdat]) |

**Supplement Table 2** Study characteristics.

| **Authors, year** | **Country** | **Study settings, design** | **Sample size** | **Gender** | **Age in years** |
| --- | --- | --- | --- | --- | --- |
| Li et al. (2020) | Afghanistan | Global School-Based Student Health Survey (GSHS) on school going adolescent, Cross sectional: stratified random sampling | 1,490 | 37.1% male | 12-15 |
| Akhter (2019) | Bangladesh | University students, Cross sectional: judgmental sampling | 360 | 70.8% male | 20–30 |
| Al Muktadir *et al.* (2019) | Bangladesh | College and university going students, Cross sectional: systematic random sampling | 475 | 59.6% male | 18–25 |
| Banik *et al.* (2020) | Bangladesh | College going adolescents in Dhaka, Cross sectional: multi stage random sampling | 518 | 51.7% male | 10–19 |
| Bipasha & Goon (2013) | Bangladesh | Private university students in Dhaka, Cross sectional: random sampling | 1,200 | 50% male | 18–24 |
| Chowdhury (2014) | Bangladesh | Medical students in Dhaka, Cross sectional: random sampling | 107 | 49.5% male | 21.2 (±1.9) |
| Goon et al. (2014) | Bangladesh | Private university students, Cross sectional: systematic random sampling | 426 | 50% male | 21.3 (5.4)^a^ |
| Karmakar *et al.* (2016) | Bangladesh | University students in Noakhali, Cross sectional | 200 | 50% male | 18–24 |
| Khan & Uddin (2020) | Bangladesh | School going adolescents, Cross sectional: two stage stratified sampling | 2,742 | 62% male | 12–15 |
| Li *et al.* (2020) | Bangladesh | Global School-Based Student Health Survey (GSHS) on school going adolescent, Cross sectional: stratified random sampling | 2,720 | 38.4% male | 12–15 |
| Rizwan et al. (2018) | Bangladesh | Medical College students in Rangpur, Cross sectional: convenience sampling | 107 | 45.8% male | 18–25 |
| Shetu (2021) | Bangladesh | Facebook users, Web-based cross sectional: non-probability convenience sampling | 300 | 64.3% male | >18 |
| Choeda *et al.* (2021) | Bhutan | School-going adolescents, Cross sectional: systematic random sampling | 5,809 | 44% male | 13–17 |
| Sim & Laohasiriwong (2019) | Cambodia | Working people of Phnom Penh, Cross sectional: multi-stage random sampling | 749 | 49.8% male | 32.3 (11.1) ^a^ |
| Li *et al.* (2020) | China | School going student in Beijing, Shanghai, Nanjing, and Xi'an, Cross sectional: multi-stage random sampling | 1,409 | 50.5% male | 11.5 (2.0)^a^ |
| Ma et al. (2016) | China | Longtan middle school in Chongwen, Cross sectional | 201 | 48.6% male | 14.0 (1.3)^a^ |
| Qin *et al.* (2021) | China | Primary and high school going students, Cross sectional: multi-stage cluster sampling | 4,388 | 50.2% male | 13.9 (2.5)^a^ |
| Song *et al.* (2015) | China | Children living in Beijing & Linyi, Cross sectional: probability sampling | 500 | – | 6.2^a^ |
| Zhao *et al.* (2017) | China | Children and adolescent of Beijing, Shanghai, Nanjing, & Xi’an, Cross sectional: random sampling | 1,648 | 51% male | 7–16 |
| Rojekar et al. (2019) | India | School going children in Nagpur, Cross sectional | 115 | – | 8–12 |
| Faizi & Mittal (2018) | India | School going students in Aligarh, Cross sectional | 400 | – | 13–15 |
| Javalkar et al. (2015) | India | Medical students in Mangalore, Cross sectional | 430 | 43% male | 20.7 (1.7)^a^ |
| Joseph et al. (2015) | India | School going students in Mangalore, Cross sectional: random sampling | 300 | 100% male | 12–16 |
| Khan et al. (2022) | India | School going students in Nagpur, Cross sectional: purposive sampling | 774 | 48.2% male | 12.8^a^ |
| Mahajan & Gothankar (2020) | India | Private colleges in Pune, Cross sectional | 300 | – | 18–20 |
| Meena & Varma (2015) | India | School going girls in Jaipur, Cross sectional | 145 | 100% female | 14–16 |
| Moitra & Madan (2022) | India | School going adolescent in Mumbai, Cross sectional: random sampling | 712 | 51.8% male | 10–12 |
| Monika et al. (2015) | India | Post graduate students of Rajasthan university hostel in Jaipur, Cross sectional | 104 | 100% female | 20–26 |
| Nawab et al. (2016) | India | School going students in Aligarh, Cross sectional: systematic random sampling | 660 | 57.6% male | 10–16 |
| Pushkar et al. (2023) | India | Medical student in Pune, Cross sectional: Web‑based | 398 | 70.4% male | 21.8 (0.4)^a^ |
| Rani & Sathiyasekaran (2013) | India | School going adolescents in Chennai, Cross sectional: random sampling | 1,842 | 48.6% male | 12–18 |
| Rauf et al. (2020) | India | Medical students, Cross-sectional: simple random sampling | 100 | 50% male | 18–26 |
| Sharma et al. (2023) | India | Medical students in Odisha, Cross sectional | 177 | 55.4% male | 19.7 (3.3)^a^ |
| Shree et al. (2018) | India | Medical students in Patna, Cross sectional | 120 | 56.7% male | 22.9 (1.5)^a^ |
| Singh & Mishra (2014) | India | School going students in Lucknow, Cross sectional: random sampling | 100 | 56% male | 9–13 |
| Vaida (2013) | India | Adolescent students in urban area of Srinagar, Cross sectional | 80 | – | 14–19 |
| Li et al. (2020) | Indonesia | Global School-Based Student Health Survey (GSHS) on school going adolescent, Cross sectional: stratified random sampling | 8,766 | 46.2% male | 12–15 |
| Lwin et al. (2017) | Indonesia | School going student in Jakarta & Bogor, Cross sectional | 394 | 46.7% male | 9.4^a^ |
| Alimoradi et al. (2017) | Iran | High school going student in Sanandaj, Cross sectional: cluster random sampling | 553 | 51.7% male | 14–18 |
| Azadbakht & Esmaillzadeh (2012) | Iran | Students of Isfahan university of Medical Sciences, Cross sectional: cluster random sampling | 289 | 100% female | 21 (7)^a^ |
| Didarloo et al. (2022) | Iran | Students of Urmia university of Medical Sciences, Cross sectional: stratified random sampling | 229 | 28.4% male | 22.1 (3.3)^a^ |
| Jahanbakhsh & Mousanezhad (2015) | Iran | College and university going students, Cross sectional: stratified sampling | 227 | 37.4% male | 21.1^a^ |
| Mirkarimi et al. (2016) | Iran | School going students in Aliabad, cross sectional | 500 | 50% male | 16.8 (0.8)^a^ |
| Mohammadbeigi et al. (2018) | Iran | Students of two universities in Qom, Cross sectional: simple random sampling | 300 | – | 21^a^ |
| Namdar et al. (2021) | Iran | Adults living in Fasa, Cross sectional | 423 | 30.2% male | 18–65 |
| Rasouli et al. (2021) | Iran | High school students in Zanjan, Cross sectional: Cluster random sampling | 370 | 100% female | 12-15 |
| Rezaei (2017) | Iran | Adults of Yasuj, southwestern Iran, Cross sectional: random sampling | 540 | 56.3% male | 18–45 |
| Sangsefidi et al. (2020) | Iran | Adult population of Yazd, Cross sectional: cluster random sampling | 9,965 | 49.7% male | 20–70 |
| Sharifirad et al. (2013) | Iran | High school going students, Cross sectional: randomized stratified sampling | 521 | 53.2% male | 15–18 |
| Shayan-Moghadam et al. (2020) | Iran | Primary and secondary schools students of 30 provinces, Cross sectional | 3,207 | Female | 12–18 |
| Taghizadeh et al. (2021) | Iran | Overweight or obese children and adolescents referred to Shahid Bakeri Health Center in Tabriz, Cross sectional | 425 | 57.9% male | 10.6 (3.5) |
| Yarmohammadi et al. (2015) | Iran | School going students in Isfahan, Cross sectional: multistage sampling | 512 | 46.8% male | 15–18 |
| Al-Delaimy et al. (2020) | Iraq | Student of three government primary schools in Ramadi, Cross sectional: simple random sampling | 512 | 45.3% male | 6–12 |
| Li et al. (2020) | Iraq | Global School-Based Student Health Survey (GSHS) on school going adolescent, Cross sectional: stratified random sampling | 1,538 | 54.6% male | 12-15 |
| Musaiger et al. (2014) | Iraq | Adolescent in Mosul city, Iraq, Cross sectional: multistage stratified sampling | 723 | 48.4% male | 15–18 |
| Rasool & AKBAY (2021) | Iraq | Adult living in Northern Iraq, Cross sectional: simple random sampling | 380 | – | 36.1^a^ |
| Mwafi et al. (2021) | Jordan | Student of Mutah university, Cross sectional | 503 | 54.7% male | 21.6 (2.2 ^a^ |
| Smith et al. (2020) | Lebanon | Global School-Based Student Health Survey (GSHS) on school going adolescent, Cross sectional: stratified random sampling | 3,347 | – | 12–15 |
| Abdullah et al. (2016) | Malaysia | Healthy adolescents in Kelantan, Cross sectional | 454 | 45% male | 12–19 |
| Azizan et al. (2018) | Malaysia | Adult in low-income urban area, Cross sectional: simple random sampling | 1,450 | 44.3% male | >18 |
| Chong et al. (2016) | Malaysia | South East Asian Nutrition Surveys, Cross sectional: stratified random sampling | 3,350 | 50.4% male | 2–12 |
| Eng et al. (2022) | Malaysia | Low-income adults in Kuala Lumpur, Cross sectional: Stratified sampling | 2,983 | 49.8% male | 26–49 |
| Gan et al. (2019) | Malaysia | Secondary school student in Selangor, Cross sectional: probability proportional to size sampling | 421 | 41.8% male | 12–16 |
| Hatta et al. (2022) | Malaysia | Young adult, Cross sectional: convenience sampling | 405 | 15.6% male | 18–29 |
| Li et al. (2020) | Malaysia | Global School-Based Student Health Survey (GSHS) on school going adolescent, Cross sectional: stratified random sampling | 16,265 | 51.2% male | 12–15 |
| Man et al. (2021) | Malaysia | Adolescents in Peninsular and East Malaysia, Cross sectional: multiple-stage stratified cluster random sampling | 26,383 | 50.2% male | 10–18 |
| Mat et al. (2016) | Malaysia | University student, Cross sectional | 215 | 68.4% male | 18–23 |
| Syafiqah et al. (2018) | Malaysia | Students of university Sultan Zainal Abidin, Cross sectional | 320 | 22.5% male | 18–26 |
| Smith et al. (2020) | Maldives | Global School-Based Student Health Survey (GSHS) on school going adolescent, Cross sectional: stratified random sampling | 1,781 | – | 12–15 |
| Li et al. (2020) | Mongolia | Global School-Based Student Health Survey (GSHS) on school going adolescent, Cross sectional: stratified random sampling | 3,691 | 47.8% male | 12-15 |
| Bohara et al. (2021) | Nepal | Adolescent students in Kaski, Cross sectional: stratified proportionate sampling | 538 | 48% male | 14–19 |
| Poudel (2018) | Nepal | Adolescents school going students in Parsa, Cross sectional: purposive sampling | 311 | 66.9% male | 12–17 |
| Singh et al. (2021) | Nepal | School going student in Kathmandu, Cross sectional: multistage cluster random sampling | 627 | 54.1% male | 12–16 |
| Smith et al. (2020) | Nepal | Global School-Based Student Health Survey (GSHS) on school going adolescent, Cross sectional: stratified random sampling | 4,616 | – | 12–15 |
| Ahmed et al. (2016) | Pakistan | High school students in Hyderabad, Cross sectional: simple random sampling | 501 | 50.9% male | 13.8 (1.6)^a^ |
| Akhtar et al. (2018) | Pakistan | Medical college students in Lahore, Cross sectional: purposive sampling | 114 | 100% Female | 19.5 (2.1)^a^ |
| Ali & Lee (2019) | Pakistan | Adult living in Lahore, Cross sectional: random sampling | 273 | 61.9% male | Greater than 20 |
| Asghar et al. (2019) | Pakistan | Medical college students in Karachi, Cross sectional: Non-probability convenient sampling | 351 | 26.5% male | 21.3 (1.5)^a^ |
| Hayyat et al. (2019) | Pakistan | School going children in Lahore, Cross sectional: random sampling | 240 | 50% male | 10–16 |
| Irfan et al. (2019) | Pakistan | University students in Gujrat, Cross sectional: random sampling | 432 | 20.9% male | 21.8 (2.9)^a^ |
| Ismail (2016) | Pakistan | Three different University students in Karachi, Cross sectional: purposive sampling | 50 | 100% Female | 19–30 |
| Mushtaq et al. (2011) | Pakistan | Student of primary schools in Lahore, Cross sectional: stratified multistage random cluster sample | 1,860 | 52.5% male | 5–12 |
| Shami & Fatima (2017) | Pakistan | College going student in Karachi, Cross sectional: convenient sampling | 50 | 100% Female | 17.3^a^ |
| Tariq et al. (2019) | Pakistan | School and college going student in Faisalabad, Cross sectional: multistage sampling | 226 | 41.5% male | 13–19 |
| Li et al. (2020) | Philippines | Global School-Based Student Health Survey (GSHS) on school going adolescent, Cross sectional: stratified random sampling | 6,163 | 43.6% male | 12–15 |
| Kim et al. (2019) | South Korea | Korea National Health and Nutrition Examination Survey 2010–2014, Cross sectional: multistage stratified clustered rolling sampling | 19,017 | – | 19–64 |
| Aruppillai & Godwin Phillip (2015) | Sri Lanka | People of Moratuwa municipal council area, Cross sectional: random sampling | 120 | – | > 5 |
| Jayasinghe & De Silva (2014) | Sri Lanka | Sri Lankan university students, Cross sectional: random sampling | 205 | 38% male | Male:  23.4 (1.2)^a^ Female:  23.2 (1.2)^a^ |
| Smith et al. (2020) | Sri Lanka | Global School-Based Student Health Survey (GSHS) on school going adolescent, Cross sectional: stratified random sampling | 2,254 | – | 12–15 |
| Alhashemi et al. (2022) | Syria | Medical students of Aleppo university, Cross sectional: simple random sampling | 514 | 46.3% male | 18–27 |
| Li et al. (2020) | Syria | Global School-Based Student Health Survey (GSHS) on school going adolescent, Cross sectional: stratified random sampling | 2,914 | 40% male | 12–15 |
| Musaiger & Kalam (2014) | Syria | School and college going adolescent, Cross-sectional multi-stage stratified sampling | 365 | 48.8% male | 15–18 |
| Khaipetch (2017) | Thailand | People of Pathum Thani area, Cross sectional: non-probability sampling | 200 | 42% male | >35 |
| Li et al. (2020) | Thailand | Global School-Based Student Health Survey (GSHS) on school going adolescent, Cross sectional: stratified random sampling | 4,109 | 47% male | 12–15 |
| Li et al. (2020) | Timor-Leste | Global School-Based Student Health Survey (GSHS) on school going adolescent, Cross sectional: stratified random sampling | 1,668 | 41.8% male | 12–15 |
| Arslan et al. (2023) | Turkey | University students in Mardin, Cross sectional: random sampling | 184 | 47.8% male | 21.1 ^a^ |
| Li et al. (2020) | Vietnam | Global School-Based Student Health Survey (GSHS) on school going adolescent, Cross sectional: stratified random sampling | 1,738 | 46.6% male | 12–15 |
| Nguyen et al. (2022) | Vietnam | Student of junior high schools in Ho Chi Minh City, Cross sectional: multistage cluster sampling | 2,660 | 50.1% | 12–14 |
| Phan & Mai (2016) | Vietnam | University students in Binh Duong, Cross sectional: convenient sampling | 100 | – | 18–23 |
| Tayem et al. (2012) | West Bank | Undergraduate student of Al-Quds University, Cross sectional: random sampling | 553 | 59.5% male | 21^a^ |
| Smith et al. (2020) | Yemen | Global School-Based Student Health Survey (GSHS) on school going adolescent, Cross sectional: stratified random sampling | 1,553 | – | 12–15 |

^a^mean

**Supplemnatry Table 3** Fast food consumption pattern among children and adolescents

| **Authors, Year** | **Country** | **Study settings; Sample size; Age; Gender** | **Fast food consumption patterns** |
| --- | --- | --- | --- |
| Li et al. (2020) | Afghanistan | Global School-Based Student Health Survey on school going adolescent; 1,490; 12-15 years; 37.1% male | • None: 37%  • 1-3 days/week: 52.2%  • 4-7 days/week: 10.8%  • ≥1 times/week: 65%  • Mean day/week: 2.4 |
| Banik et al. (2020) | Bangladesh | College going adolescents in Dhaka; 518; 10–19 years; 51.7% male | • Once/week: 68.3%  • 1-3 days/week: 35.9% • >3 days/week: 64.1%  • Male consumed more than female |
| Khan & Uddin, (2020) | Bangladesh | School going adolescents; 2,742; 12-15 years; 62% male | • 1-2 days/ week: 28%  • ≥3 days/ week: 26% |
| Li et al. (2020) | Bangladesh | Global School-Based Student Health Survey on school going adolescent; 2,720; 12-15 years; 38.4% male | • None: 46.6%  • 1-3 days/week: 42.9%  • 4-7 days/week: 10.5%  • ≥1 times/week: 53.2%  • Mean day/week: 2.4 |
| Choeda et al. (2021) | Bhutan | School-going adolescents; 5,809; 13–17 years; 44% male | • Weekly consumption: 90.4% |
| Li et al. (2017) | China | School going student in Beijing, Shanghai, Nanjing, and Xi'an; 1,409; 11.5 (2) years ^a^; 50.5% male | • Mean consumption/ week: 0.6 (1)  • Male were significantly frequent consumer than female |
| Ma et al. (2016) | China | Longtan middle school in Chongwen; 201; 14.0 (1.3) years ^a^; 48.6% male | • Mean consumption/ week: 1.5 (1.3) |
| Qin et al. (2021) | China | Primary and high school going students; 4,388; 13.9 (2.5) years ^a^; 50.2% male | • Weekly consumption: 32.4% |
| Zhao et al. (2017) | China | Children and adolescent of Beijing, Shanghai, Nanjing, & Xi’an; 1,648; 7–16 years; 51% male | • Western fast food/week: 51.9%  • Chinese fast food/week: 43.6% |
| Rojekar et al. (2019) | India | School going children in Nagpur; 115; 8–12 years; NA | • 2 times/week: 90% |
| Faizi & Mittal (2018) | India | School going students in Aligarh; 400; 13–15 years; NA | • Once/week: 28.8%  • 2-3 times/ week: 36.4% • ≥4 times/week: 21.1% |
| Joseph et al. (2015) | India | School going students in Mangalore; 300; 12–16 years; All male | Fast food habit: 97.3%  • Once/week: 62.7%  • 2 times/week: 15.4%  • >2 times/week: 21.9% |
| Khan et al. (2022) | India | School going students in Nagpur; 774; 12.8 years; 48.2% male | • ≥3 days/week: 90.4% |
| Nawab et al. (2016) | India | School going students in Aligarh; 660; 10–16 years; 57.6% male | • Occasionally: 17%  • 1-2 times/week: 44.2%  • ≥3 times/week: 38.8% |
| Rani & Sathiyasekaran (2013) | India | School going adolescents in Chennai; 1,842; 12–18 years; 48.6% male | • Never: 15% • 1-3 times/week: 62.6% • 4-7 times/week: 22.4% |
| Singh & Mishra (2014) | India | School going students in Lucknow; 100; 9–13 years; 56% male | • 98% preferred fast food |
| Li et al. (2020) | Indonesia | Global School-Based Student Health Survey on school going adolescent; 8,766; 12–15 years; 46.2% male | • None: 44.2%  • 1-3 days/week: 45.3%  • 4-7 days/week: 10.5%  • ≥1 times/week: 56.5%  • Mean day/week: 2.3 |
| Alimoradi et al. (2017) | Iran | High school going student in Sanandaj; 553; 14–18 years; 51.7% male | • Once/week: 69.8% • >3 times/week among boys: 9.9%  • >3 times/week among girls: 9.8% |
| Rasouli et al. (2021) | Iran | High school students in Zanjan; 370; 12–15 years; All female | • None: 7%  • Daily: 2.4%  • Weekly: 22.7%  • Monthly: 43.9%  • Seasonally: 13.8%  • Yearly: 10.8% |
| Shayan-Moghadam et al. (2020) | Iran | Primary and secondary schools students of 30 provinces; 3,207; 12–18 years; All female | Fast food:  • Never: 18.2%  • Rarely: 55%  • Daily: 10.6%  • Weekly: 16.2%  Sweet snacks:  • Never: 2.5%  • Rarely: 34.4%  • Daily: 40.8%  • Weekly: 22.3% |
| Taghizadeh et al. (2021) | Iran | Overweight or obese children and adolescents referred to Shahid Bakeri Health Center in Tabriz; 425; 10.6 (3.5) years ^a^; 57.9% male | • Rarely: 67.1%  • Daily: 8.7%  • 1–2 items/week: 24.2 |
| Yarmohammadi et al. (2015) | Iran | School going students in Isfahan; 512; 15–18 years; 46.8% male | • Once/month: 26.3% • 2 times/month: 26.9% • 1-3 times/week:12.5%  • ≥3 times/ week: 2.7% |
| Al-Delaimy et al. (2020) | Iraq | Student of three government primary schools in Ramadi; 512; 6–12 years; 45.3% male | • Once/week: 34.6% • 2-3 times/week: 12.3%  • Daily: 1.8% • Never: 51.4% |
| Li et al. (2020) | Iraq | Global School-Based Student Health Survey on school going adolescent; 1,538; 12–15 years; 54.6% male | • None: 44.2%  • 1-3 days/week: 45.3%  • 4-7 days/week: 10.5%  • ≥1 times/week: 56.5%  • Mean day/week: 2.3 |
| Musaiger et al. (2014) | Iraq | Adolescent in Mosul city, Iraq; 723; 15–18 years; 48.4% male | Mean consumption/ week-  • Male: 3.0 (2.2) times  • Female: 2.4 (2.0) times  >3 days/week-  • Male: 37.1%  • Female: 24.9 % |
| Smith et al. (2020) | Lebanon | Global School-Based Student Health Survey on school going adolescent; 3,347; 12–15 years; NA | • Weekly consumption: 77.1% |
| Abdullah et al. (2016) | Malaysia | Healthy adolescents in Kelantan; 454; 12–19 years; 45% male | • Mean weekly consumption 1.1 times |
| Chong et al. (2016) | Malaysia | South East Asian Nutrition Surveys; 3,350; 2–12 years; 50.4% male | • ≥1 times/week: 9.7% |
| Gan et al. (2019) | Malaysia | Secondary school student in Selangor; 421; 12–16 years; 41.8% male | • None: 21%  • 1-2 times/week: 52.1%  • ≥3 times/week: 26.9% |
| Li et al. (2020) | Malaysia | Global School-Based Student Health Survey on school going adolescent; 16,265; 12–15 years; 51.2% male | • None: 51.7%  • 1-3 days/week: 45.9%  • 4-7 days/week: 2.4%  • ≥1 times/week: 47.8%  • Mean day/week: 1.8 |
| Man et al. (2021) | Malaysia | Adolescents in Peninsular and East Malaysia; 26,383; 10–18 years; 50.2% male | • 1–3 days: 69.3%  • 4-7 days/weeks: 13.5%  • Female consumed more than male |
| Smith et al. (2020) | Maldives | Global School-Based Student Health Survey on school going adolescent; 1,781; 12–15 years; NA | • Weekly consumption: 35.1% |
| Li et al. (2020) | Mongolia | Global School-Based Student Health Survey on school going adolescent; 3,691; 12–15 years; 47.8% male | • None: 44.9%  • 1-3 days/week: 37.7%  • 4-7 days/week: 17.4%  • ≥1 times/week: 55.1%  • Mean day/week: 2.6 |
| Bohara et al. (2021) | Nepal | Adolescent students in Kaski; 538; 14–19 years; 48% male | • Monthly junk foods consumption: 60.3% |
| Poudel (2018) | Nepal | Adolescents school going students in Parsa; 311; 12–17 years; 66.9% male | • Daily: 33.8%  • One time/ week: 22.2%  • 2 times/ week: 24.8%  • >2 times: 19.3% |
| Singh et al. (2021) | Nepal | School going student in Kathmandu; 627; 12–16 years; 54.1% male | • Daily: 10.3%  • 2–3 days/week: 53.4%  • ≥ 4 days/week: 36.2% |
| Smith et al. (2020) | Nepal | Global School-Based Student Health Survey on school going adolescent; 4,616; 12–15 years; NA | • Weekly consumption: 75.3% |
| Ahmed et al. (2016) | Pakistan | High school students in Hyderabad; 501; 13.8 (1.6) years^a^; 50.9% male | • Never: 56%  • 1-3 times/week: 44% |
| Hayyat et al. (2019) | Pakistan | School going children in Lahore; 240; 10–16 years; 50% male | • 1-2 times/week:72.9% • 3-4 times/week:17.9% • Daily: 5.4% |
| Mushtaq et al. (2011) | Pakistan | Student of primary schools in Lahore; 1,860; 5–12 years; 52.5% male | • Occasionally: 57.1%  • 1-2 times/week: 29.5%  • ≥3 times/week: 13.4%  • Male consumed more fast foods than female |
| Shami & Fatima (2017) | Pakistan | College going student in Karachi; 50; 17.3 years^a^; All female | Everyone consumed fast food  Frequent consumer: 54%  • Daily: 8%  • 2-3 times/week: 20%  • Once/week: 26%  Infrequent consumer: 46% |
| Tariq et al. (2019) | Pakistan | School and college going student in Faisalabad; 226; 13–19 years; 41.5% male | • Male consumed 2.1 (2.0) times/week  • Female consumed 2.5 (2.4) times/week |
| Li et al. (2020) | Philippines | Global School-Based Student Health Survey on school going adolescent; 6,163; 12–15 years; 43.6% male | • None: 48.1%  • 1-3 days/week: 47.8%  • 4-7 days/week: 4.1%  • ≥1 times/week: 49%  • Mean day/week: 1.9 |
| Aruppillai & Godwin Phillip (2015) | Sri Lanka | People of Moratuwa municipal council area; 120; <15 years; NA | • Daily: 20%  • Once/week: 60%  • 1-2 times/ month: 20% |
| Smith et al. (2020) | Sri Lanka | Global School-Based Student Health Survey on school going adolescent; 2,254; 12–15 years; NA | • Weekly consumption: 42.8% |
| Musaiger & Kalam (2014) | Syria | School and college going adolescent; 365; 15–18 years; 48.8% male | • <4 times/week: 85.8%  • ≥4 times/week: 14.2%  • Male consumed more frequently than female |
| Li et al. (2020) | Syria | Global School-Based Student Health Survey on school going adolescent; 2,914; 12–15 years; 40% male | • Never: 57.2%  • 1-3 times/week: 37.8%  • 4-7 days/week: 5%  • ≥1 times/week: 42.5%  • Mean day/week: 1.8 |
| Li et al. (2020) | Thailand | Global School-Based Student Health Survey on school going adolescent; 4,109; 12–15 years; 47% male | • None: 19.9  • 1-3 days/week: 36.8  • 4-7 days/week: 43.3  • ≥1 times/week: 81.8%  • Mean day/week: 4.1 |
| Li et al. (2020) | Timor-Leste | Global School-Based Student Health Survey on school going adolescent; 1,668; 12–15 years; 41.8% male | • None: 32.8%  • 1-3 days/week: 55.4%  • 4-7 days/week: 11.8%  • ≥1 times/week: 64.9%  • Mean day/week: 2.4 |
| Li et al. (2020) | Vietnam | Global School-Based Student Health Survey on school going adolescent; 1,738; 12–15 years; 46.6% male | • None: 70.3%  • 1-3 days/week: 24.4%  • 4-7 days/week: 5.3%  • ≥1 times/week: 30.3%  • Mean day/week: 1.7 |
| Nguyen et al. (2022) | Vietnam | Student of junior high schools in Ho Chi Minh City; 2,660; 12–14 years; 50.1% male | • Occasionally/month: 16.1%  • Once/month to once/week: 82.3%  • 2-6 times/week: 1.6%  • Male consumed more than female |
| Smith et al. (2020) | Yemen | Global School-Based Student Health Survey on school going adolescent; 1,553; 12–15 years; NA | • Weekly consumption: 34.5% |

**Supplementary Table 4** Fast food consumption pattern among adults.

| **Author Name** | **Country** | **Study settings; Sample size; Age; Gender** | **Fast food consumption patterns** |
| --- | --- | --- | --- |
| Akhter (2019) | Bangladesh | University students; 360; 20–30 years; 70.8% male | Fast food preference: 94.2%  • Preference of foreign fast-food restaurants: 60.3% • Preference of local fast-food restaurants: 39.7%  • 1-3 times/month: 48.1% • >4 times/month: 45.6% |
| Al Muktadir et al. (2019) | Bangladesh | College and university going students; 475; 18-25 years; 59.6% male | • Once/week: 40.8%  • 2 times/week: 27.2%  • ≥4 times/week: 4.6% |
| Bipasha & Goon (2013) | Bangladesh | Private university students in Dhaka; 1200; 18-24 years; 50% male | • Weekly consumption: 98.5%  • Male preferred fast foods over female |
| Chowdhury (2014) | Bangladesh | Medical students in Dhaka; 107; 21.2 (±1.9) years; 49.5% male | • Daily: 15%  • Once/week: 50.5%  • Rarely: 34.6% |
| Goon et al. (2014) | Bangladesh | Private university students; 426; 21.3 years; 50% male | Fast-food consumption/week: 54%  • Male: 55.9%  • Female: 44.1% |
| Karmakar et al. (2016) | Bangladesh | University students in Noakhali; 200; 18–24; 50% male | • Junk food preference: 70.5%  • 1-2 times/week: 24% • ≥2 times/week: 41 % |
| Rizwan et al. (2018) | Bangladesh | Medical College students in Rangpur; 107; 18–25 years; 45.8% male | • Never: 34%  • Once/month: 25.2%  • 2-3 times/month: 29%  • ≥4 times/month: 14% |
| Shetu (2021) | Bangladesh | Facebook users, 300; >18 years; 64.3% male | • Once/month: 20.1% • 1-3 times/month: 44.5% • 3-5 times/month: 18.1% • >5 times/month: 17.3% |
| Sim & Laohasiriwong (2019) | Cambodia | Working people of Phnom Penh; 749; 32.3 (11.1) years; 49.8% male | • Monthly fast food consumption: 62.7% |
| Javalkar et al. (2015) | India | Medical students in Mangalore; 430; 20.7 (1.7) years; 43% male | • 1-2 times/week: 42.8% • 3-4 times/week: 23.7% • Daily: 19.1% |
| Mahajan & Gothankar (2020) | India | Private colleges in Pune; 300; 18–20 years; NA | • 85% consumed fast foods  • Once/day: 17% |
| Pushkar et al. (2023) | India | Medical student in Pune; 398; 21.8 (0.4) years; 70.4% male | Everyone consumed fast food  • <3 times/week: 80.4%  • ≥3 times/week: 19.6% |
| Rauf et al. (2020) | India | Medical students; 100; 18–26 years; 50% male | • 1-2 times/week: 65%  • ≥3 times/week: 30% |
| Sharma et al. (2023) | India | Medical students in Odisha; 177; 19.7 (3.3) years; 55.4% male | • <1 time/week: 6.8%  • 3-4 times/week: 30%  • >5 times/week: 44.6% |
| Shree et al. (2018) | India | Medical students in Patna; 120; 22.9 (1.5) years; 56.7% male | • <1 times/week: 10%  • 1-2 times/ week: 75.6%  • 3-4 times/ week: 13.3  • >5 times/ week: 8.3% |
| Azadbakht & Esmaillzadeh (2012) | Iran | Students of Isfahan university of Medical Sciences; 289; 21 (7) years; 100% female | • Occasionally: 50%  • 2 times/week: 42%  • >2 times/week: 8% |
| Didarloo et al. (2022) | Iran | Students of Urmia university of Medical Sciences; 229; 22.1 (3.3) years; 28.4% male | • Monthly mean consumption: 2.7 times  • Male consumed more than female |
| Jahanbakhsh & Mousanezhad (2015) | Iran | College and university going students; 227; 21.1 years; 37.4% male | • Once/month: 15.9%,  • 2 times/month: 25.6%,  • 1-2 times/week:20.3%  • 3-5 times/week: 7.5% • Once/day: 3.1 |
| Mohammadbeigi et al. (2018) | Iran | Students of two universities in Qom; 300; 21 years; NA | Monthly fast food consumption: 72.4%  • Males: 80.7%  • Females: 67.4% |
| Namdar et al. (2021) | Iran | Adults living in Fasa; 423; 18–65 years; 30.2% male | • 1–2 times/week: 25.4%  • >2 times/week: 3.8% • 1-2 times/month: 20.9% • Occasionally: 49.9% |
| Rezaei (2017) | Iran | Adults of Yasuj, southwestern Iran; 540; 18–45 years; 56.3% male | • Once/month: 8.14%,  • 2 times/month: 11.8% • Once/week: 16.8% • 2 times/week: 28.8%  • 3-6 times/week: 18.1% • Once/day: 5% |
| Sangsefidi et al. (2020) | Iran | Adult population of Yazd; 9,965; 20–70 years; 49.7% male | • Few times/years: 60.2%  • 1–3 times/month: 29.1%  • Once or more/week: 10.7% |
| Mwafi et al. (2021) | Jordan | Student of Mutah university; 503; 21.6 (2.2) years; 54.7% male | • ≥2 times/week: 59.4% |
| Azizan et al. (2018) | Malaysia | Adult in low-income urban area; 1,450; >18 years; 44.3% male | • ≥4 times/month: 24.3%  • <4 times/month: 75.7% |
| Eng et al. (2022) | Malaysia | Low-income adults in Kuala Lumpur; 2,983; 26–49 years; 49.8% male | • ≥Once/day: 0.9%  • ≥Once/week: 15%  • Seldom/never: 84.1% |
| Mat et al. (2016) | Malaysia | University student; 215; 18–23 years; 68.4% male | Male:  • 1-2 times/month: 50%  • 1-2 times/week: 17.6%  • >3 times/week: 5.9% Female:   • 1-2 times/month: 36.1%  • 1-2 times/week: 27.2%  • >3 times/week: 6.8% |
| Syafiqah et al. (2018) | Malaysia | Students of university Sultan Zainal Abidin; 320; 18–26 years; 22.5% male | • 93% preferred fast food • 1-5 times/week: 99% |
| Akhtar et al. (2018) | Pakistan | Medical college students in Lahore; 114; 19.5 (2.1) years; 100% Female | • 64% consumed fast food |
| Ali & Lee (2019) | Pakistan | Adult living in Lahore; 273; >20 years; 61.9% male | • Occasionally: 48.71% • Weekends: 26.7% • Seldom: 16.1% |
| Asghar et al. (2019) | Pakistan | Medical college students in Karachi; 351; 21.3 (1.5) years 26.5% male | • None: 8.8%  • 1-2 times/week: 62.6%  • ≥3 times/week: 28.6 |
| Irfan et al. (2019) | Pakistan | University students in Gujrat; 432; 21.8 (2.9) years; 20.9% male | • 1-3 times/week: 26.2%  • 4-6 times/week: 26.2%  • 1-2 times/daily: 5.8% |
| Ismail (2016) | Pakistan | Three different University students in Karachi; 50; 19–30 years; 100% Female | 94% consumed fast food  Frequent consumer: 57.4%  • Daily: 17%  • 2-3 times/week: 14.9%  • Once/week: 25.5%  Infrequent consumer: 42.5% |
| Kim et al. (2019) | South Korea | Korea National Health and Nutrition Examination Survey 2010–2014; 19,017; 19–64 years; NA | • 10% habituated to fast foods |
| Jayasinghe & De Silva (2014) | Sri Lanka | Sri Lankan university students; 205; 23 years; 38% male | • Daily: 54% • 4-6 times/week: 12% • 2-3 times/week: 21% • Once/week: 11% |
| Aruppillai & Godwin Phillip (2015) | Sri Lanka | People of Moratuwa municipal council area; 120; >30 years; NA | 31-45 years  • Once/week: 11.1%  • 1-2 times/ month: 66.7%  ≥46 years  • Once/week: 16.7%  • 1-2 times/ month: 83.3% |
| Alhashemi et al. (2022) | Syria | Medical students of Aleppo university; 514; 18–27 years; 46.3% male | • None: 26.5%  • Once/week: 44.5%  • 2 times/week: 17.7%  • ≥3 times/week: 11.4% |
| Arslan et al. (2023) | Turkey | University students in Mardin; 184; 21.1 (6) years; 47.8% male | Female:  • Once/15 days: 40.6%  • 3 times/week: 19.8%  • 5 times/week: 11.5%  • Once/month: 28.2%  Male:  • Once/15 days: 38.6%  • 3 times/week: 28.4%  • 5 times/week: 5.6%  • Once/month: 27.3%  Significant gender variation observed |
| Phan & Mai (2016) | Vietnam | University students in Binh Duong; 100; 18–23 years; NA | • 1-2 times/week: 42%, • 1-2 times/month: 27% |
| Tayem et al. (2012) | West Bank | Undergraduate student of Al-Quds University; 553; 21 years; 59.5% male | • None: 16.6%  • 12 times/week: 30.9%  • 3 times/week: 52.4%  • Male consumed more fast food than female |
